# Pulmonary hypertension in extremely preterm infants: a call to standardize echocardiographic screening and follow-up policy

**DOI:** 10.1007/s00431-021-03931-5

**Published:** 2021-02-02

**Authors:** Sanne Arjaans, Elvira. A. H. Zwart, Marc Roofthooft, Elisabeth M. W. Kooi, Arend F. Bos, Rolf M. F. Berger

**Affiliations:** 1 Center for Congenital Heart Disease, Department of Pediatric Cardiology, Beatrix Children’s Hospital, University Medical Center Groningen, University of Groningen, Hanzeplein 1, PO Box 30.001, 9700 RB Groningen, the Netherlands; 2 Department of Neonatology, Beatrix Children’s Hospital, University Medical Center Groningen, University of Groningen, Groningen, the Netherlands

**Keywords:** Pulmonary hypertension, Bronchopulmonary dysplasia, Extremely preterm infants, Screening and follow-up

## Abstract

Pulmonary hypertension (PH) is a frequent complication in extremely preterm born infants that seriously affects outcome. We aimed to describe the prevalence of PH in extremely preterm infants and the policy on screening and follow-up in the ten Dutch intensive care units (NICUs). We performed a retrospective cohort study at the University Medical Centre Groningen on infants with gestational age < 30 weeks and/or birthweight < 1000 g, born between 2012 and 2013. Additionally, we carried out a survey among the Dutch NICUs covering questions on the awareness of PH, the perceived prevalence, and policy regarding screening and following PH in extremely preterm infants. Prevalence of early-onset PH in our study was 26% and 5% for late-onset PH. PH was associated with poor survival and early-onset PH was associated with subsequent development of bronchopulmonary dysplasia (BPD). All the NICUs completed the questionnaire and we found that no standardized policy existed regarding screening and following PH in extremely preterm infants.

*Conclusion*: Despite the frequent occurrence of PH and its clinically important consequences, (inter-)national standardized guidelines regarding screening and following of PH in extremely preterm infants are lacking. Standardizing screening and follow-up will enable early identification of infants with late-onset PH and allow for earlier treatment. Additionally, greater clarity is required regarding the prevalence of early PH as are new preventive treatment strategies to combat BPD.

**Table:** 

**What is known?** • *Pulmonary hypertension (PH) substantially impairs the survival of extremely preterm infants.* • *PH is associated with bronchopulmonary dysplasia (BPD): Early-onset PH predicts the development of BPD. Late-onset PH is prevalent in infants with severe BPD.*	
**What is new?** • *Pulmonary hypertension (PH) is prevalent in preterm infants. Its consequences for morbidity and mortality justify a standardized policy aimed at early detection to improve prevention and treatment.* • *No structured policy exists in the Netherlands regarding screening/follow-up for PH in extremely preterm infants.*

**Supplementary Information:**

The online version contains supplementary material available at 10.1007/s00431-021-03931-5.

## Introduction

During the past decades, the mortality of extremely preterm infants has decreased significantly. Nevertheless, these infants are at substantial risk for debilitating comorbidities and increased mortality in early childhood, the latter being as high as 35% for infants with a gestational age of less than 25 weeks [1–4].

Bronchopulmonary dysplasia (BPD), the chronic lung disease of infancy, remains such a complication of extreme preterm birth that, on account of infants’ increasingly younger gestational ages, is associated with very early disruption of normal development of the lungs. By international consensus, BPD is diagnosed when an infant has received oxygen supplementation for more than 28 days at 36 weeks postmenstrual age (PMA) and is classified according to the severity of the condition as mild, moderate, or severe [5, 6]. However, the chronic lung disorder of prematurity develops over time is strongly associated with pulmonary vascular disease, starts already prenatally, and evolves into childhood and adolescence. Also, severe lung disease associated with preterm birth may lead to early mortality before the designation of BPD at 36 weeks PMA. Recently, an international neonatal consortium emphasized that BPD is part of an overarching condition called chronic pulmonary insufficiency of prematurity (CPIP) and that the dichotomous definition of BPD (yes/no) is insufficient to cover the spectrum and hampers the development of new therapies. Identification of early risk factors for late cardiorespiratory outcomes is required to design and install personalized preventive treatment strategies [7]. Despite improvements in ventilation therapies, including surfactant therapy, non-invasive ventilation techniques, and other improvements in perinatal care, the prevalence of BPD in extremely preterm infants rises currently up to 40% [1]. Pulmonary hypertension (PH), an increased pressure in the pulmonary arteries, develops in 20–40% depending on the severity of BPD and may lead to right-sided heart failure and death. The presence of PH in infants with BPD contributes substantially to morbidity and mortality [8–13]. Also, PH may present early after birth, before 36 weeks PMA, and thus before a diagnosis of BPD.

PH is often arbitrarily divided into early-onset PH and late-onset PH. Early-onset PH occurs at birth or during the first weeks of life and may have very different pathophysiologies with either increased pulmonary vascular resistance (PVR), such as persistent neonatal PH (PPHN), or with postnatal decreased PVR, such as PH associated with increased pulmonary blood flow due to left-to-right shunting through a patent ductus arteriosus (PDA). Late-onset PH develops at a later stage and is often referred to as PH associated with BPD from the age of 36 weeks PMA.

The occurrence of PH in extremely preterm infants, either early or late, is associated with a significant decrease in survival. For example, approximately 20% of infants with PPHN dies before discharge [14], while late-onset PH is associated with a 48% mortality in the first 2 years following PH diagnosis [10, 15–18]. These impressive figures call for an active policy to early detect PH and its possible risk factors, to define its evolution over time in order to better understand CPIP and to facilitate the development of preventive and therapeutic measures. Although PH is recognized as an underdiagnosed condition in infants with BPD, the recent ERS guidelines for BPD and neonatal outpatient follow-up lack clear recommendations for systematic, uniform screening, and follow-up of PH in preterm infants, with or without BPD [19]. Our aim was to assess the current clinical practice regarding the screening and follow-up of PH in extremely preterm born infants and to assess to what extent this is standardized.

## Methods

### Retrospective cohort study

#### Study design and included infants

We included all preterm infants with a gestational age of less than 30 weeks and/or with a birthweight of less than 1000 g who had been admitted to the NICU of University Medical Center Groningen (UMCG), the Netherlands, between 2012 and 2013.

#### Data collection

Clinical data concerning pregnancy, birth, and outpatient follow-up were collected for each patient. Echocardiographic assessments were performed on clinical indications, such as suspected PDA, suspected PH, circulatory failure, or clinical deterioration. Regarding mortality, the death dates of the included infants were recorded or we established whether the infants were still alive after 2 years of outpatient follow-up. For this study, the timepoint of echocardiographic assessment was assigned to one of three-time slots: (a) the first 2 weeks after birth (early), (b) the period between 2 weeks after birth to 36 weeks PMA (intermediate), and (c) at/after 36 weeks PMA (late). Data are managed using a REDCap (Research Electronic Data Capture) database hosted at the University of Groningen [20].

#### Definitions of PH

Early-onset PH was PH diagnosed in the first 2 weeks after birth and included PPHN, defined as the presence of a (predominantly) right-to-left shunt through an open foramen ovale (OFO) and/or PDA, determined by echocardiography and/or clinically by a preductal and postductal difference in transcutaneous oxygen saturation (tcSO_2_) of more than 10% [21, 22].

Flow-associated PH (flow PH) is defined as a nonrestrictive (Vmax < 2.0 m/s) (predominantly) left-to-right shunt through an PDA as revealed by echocardiography.

Late-onset PH was PH diagnosed at or from 36 weeks PMA and defined as the echocardiographic presence of one of the following variables [8, 22, 23]: an estimated systolic right ventricular pressure (RVSP) of more than 40 mm Hg, RVSP/systolic blood pressure of more than 0.5, the presence of a right-to-left shunt in case of persistent duct, or any degree of systolic flattening of the interventricular septum.

#### Definition of BPD

BPD was defined as requiring oxygen (FiO_2_ > 21%) for at least 28 days at 36 weeks PMA [5]. If infants had already been transferred to another hospital by this age, it was determined whether they had met the previously mentioned BPD criteria before discharge from the NICU and/or whether the presence of BPD had been confirmed during outpatient follow-up.

#### Statistical analyses

The patient characteristics of the population and the prevalence of the different types of PH in the designated time slots were described with descriptive statistics. Data are presented, depending on the distribution of the variable, as mean ± SD, median and interquartile range (IQR), or frequencies with percentages. All tests were two-sided and *P* values < 0.05 considered significant. Patient characteristics were compared using Student’s *t* test, Mann-Whitney test, or chi-square test as appropriate. Thereafter, univariate regression was performed to determine associations of BPD and/or PH. In addition, univariate and bivariate logistic regressions were used to determine the association between early-onset PH and the subsequent development of BPD and/or late-onset PH at 36 weeks PMA. Kaplan-Meier curves were used to depict the survival of the preterm infants and a log-rank test was used to compare the different groups. All statistical analyses were performed using IBM SPSS Statistics for Windows, Version 23.0, and R and R studio version 1.14 statistical software [24–26].

### Survey among the Dutch NICUs

In July 2017, we sent a questionnaire to a representing neonatologist of each of the ten NICUs in the Netherlands. To assess the center’s awareness of PH in preterm infants and its prevalence, the representatives were asked to estimate how often BPD and PH occurred among preterm infants treated at their centers. They were also asked what policy was in place at their center for screening and follow-up of PH in this population (Table 1). The survey was designed by one author of the current study. Two other authors have reviewed the survey on clarity and made changes accordingly. The survey was sent to all ten Dutch NICUs using the personal email address of the representative neonatologist of each NICU. The questionnaire was created and carried out using a RedCap database [20].

**Table 1:** Questionnaire

Question	Answer options
*The questions below relate to the clinical practice in your NICU between 2012 and 2015*	
How many infants (< 32 weeks) are admitted to your NICU annually?	Please provide an estimate
What percentage of these infants (< 32 weeks) develops BPD?	Please provide an estimate
What percentage of these infants with BPD (< 32 weeks) develops PH at 36 weeks PMA?	Please provide an estimate
What percentage of all infants without BPD (< 32 weeks) develops PH at 36 weeks PMA?	Please provide an estimate
*Screening policy at your NICU between 2012 and 2015*	
Does your NICU have a standardized policy regarding screening and/or follow-up of PH in preterm infants (< 32 weeks)?	- Yes - No
- Describe the clinical practice for diagnosing PH in preterm infants (< 32 weeks) in your NICU	Give a short description
Does your NICU perform a standard echocardiographic assessment looking for signs of PH in preterm infants (< 32 weeks) during the first two weeks after birth?	- Yes, this is standard procedure in all preterm infants (< 32 weeks) - Yes, only on clinical indication - No
- Which clinical conditions are involved?	List the most common clinical indications
Does your NICU perform a standard echocardiographic assessment looking for signs of PH in preterm infants (< 32 weeks) at 36 weeks PMA?	- Yes, this is standard procedure in all preterm infants (< 32 weeks) - Yes, only on clinical indication - No
- Which clinical conditions are involved?	List the most common clinical indications here
Does your NICU perform a standard echocardiographic assessment looking for signs of PH in preterm infants (< 32 weeks) between 36 weeks PMA and 2 years?	- Yes, this is standard procedure in all preterm infants (< 32 weeks) - Yes, this is standard procedure in all preterm infants with BPD - Yes, only on clinical indication - No
- Which clinical conditions are involved?	List the most common clinical indications here
Is the presence of echocardiographic signs of PH during the first two weeks after birth reason for standard echocardiographic follow-up on your NICU?	- Yes - No
At what ages is this performed?	Give a short description
When is echocardiographic follow-up performed?	More than one answer possible - In case of a clinical deterioration - In case of no clinical improvement - Other
- Other reasons are:	Give a short description
What is the echocardiographic definition of PH adhered to in your NICU?	Give a short description
Has the policy on screening and follow-up of PH in preterm infants (< 32 weeks) in your NICU changed after 2015?	- Yes - No
- What changes have been made to your NICU?	Give a short description

Contents of questionnaire completed by the Dutch centers. *NICU*, neonatal intensive care unit; *BPD*, bronchopulmonary dysplasia; *PH*, pulmonary hypertension; *PMA*, postmenstrual age

## Results

### Retrospective cohort study

**Fig. 1:**
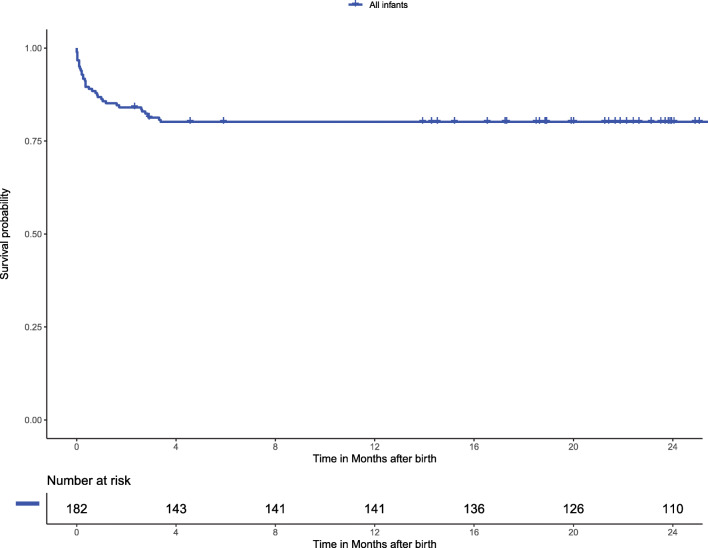
Kaplan-Meier curve showing the survival curve of all included infants. In addition, a table of the number of infants at risk is shown

One hundred eighty-two infants were included in the study and the median follow-up was 26.8 months (IQR 15.0–28.8). Thirty-six infants died during follow-up (Fig. 1). Of the 146 surviving infants, 23 were followed for less than 24 months. These latter infants had a median follow-up of 17.2 months (IQR 13.9–19.9) and had more often chorioamnionitis and were more from multiple gestations, when compared to infants followed ≥ 24 months (supplementary Table 1). The patient characteristics are shown in Tables 2 and 3. During follow-up, a diagnosis of necrotizing enterocolitis, intraventricular hemorrhage, and retinopathy of prematurity was made in 18%, 20%, and 7% of the infants respectively. In the total study period, PH was diagnosed in 56 of the 182 infants (31%). In the first 2 weeks after birth, 118 of 182 (65%) infants were echocardiographically assessed for PH and early-onset PH was diagnosed in 48 infants. Between the second week after birth and 36 weeks PMA, 77 of 163 children (47%) were echocardiographically assessed and PH was diagnosed in 17 infants. At/after 36 weeks PMA, 33 of the 153 infants (22%) were echocardiographically assessed and late-onset PH was diagnosed in 8 infants. Fifty-three infants (35%) developed BPD. Of these, 19 (36%) were echocardiographically assessed for PH (Table 4). A detailed overview of the time of PH diagnosis and the phenotyping of PH is shown in supplementary Figure 1.

**Table 2:** Patient characteristics

Variable	All *N* = 182	BPD, *n* = 53	No BPD, *n* = 100	*P* value	OR (95% CI)	*P* value
Sex (male), *n* (%)	97 (54%)	31 (58.5%)	47 (47%)	.23	-	-
Gestational age, weeks	27.3 ± 1.5	26.9 ± 1.3	28.0 ± 1.1	< .001	0.5 (0.3–0.7)	< .001
Birthweight, g	1029.6 ± 271.2.8	874.0 ± 176.69	1156.8 ± 265.9	< .001	0.995 (0.993–0.997)	< .001
Apgar score, 5th minute	7.00 (6.0- 8.0)	7.0 (5.0 - 8.0)	7.0 (6.0 - 8.0)	*.017*	0.8 (0.6–0.9)	.018
Cesarean section, *n* (%)	92 (50%)	32 (60.4%)	49 (49%)	.23	-	-
Multiple gestation, *n* (%)	43 (25%)	9 (17%)	29 (29%)	.069	-	-
Antenatal corticosteroids, *n* (%)	144 (79.1%)	42 (79.2%)	83 (83.0%)	.66	-	-
IUGR, *n* (%)	33 (18%)	17 (32.1%)	12 (12.0%)	.004	3.5 (1.5–8.0)	.004
PPROM, *n* (%)	33 (18%)	8 (15.1%)	16 (16%)	1.00	-	-
Clinical chorioamnionitis, *n* (%)	4 (2.2%)	0 (0%)	2 (2%)	.54	-	-
Antepartum bleeding, *n* (%)	18 (10%)	2 (3.8%)	10 (10%)	.22	-	-
Oligohydramnios, *n* (%)	14 (8%)	2 (3.8%)	6 (6%)	.72	-	-
Preeclampsia, *n* (%)	21 (12%)	14 (26.4%)	5 (5.0%)	< .001	6.8 (2.3–20.2)	.001

The table shows the patient characteristics of all infants and of the sub-cohorts with and without BPD. Data are presented, depending on the distribution of the variable, as mean ± SD, median and interquartile range, or frequencies with percentages. Odds ratios are shown for variables with a significant association with BPD. *IUGR*, intra-uterine growth retardation; *PPROM*, prolonged premature rupture of membranes; *BPD*, bronchopulmonary dysplasia; *OR*, odds ratio; *CI*, confidence interval

**Table 3:** Patient characteristics infants with and those without late-onset PH

Variable	Late-onset PH, *n* = 8	No late-onset PH, *n* = 25	*P* value	OR (95% CI)	*P* value
Sex (male), *n* (%)	6 (75%)	10 (40%)	.12	-	-
Gestational age, weeks	26.8 ± 1.3	27.2 ± 1.4	.43	-	-
Birthweight, g	769.4 ± 173.1	1032.6 ± 195.4	.002	0.991 (0.984–0.998)	.017
Apgar score, 5th minute	7.00 (5.0–9.0)	7.0 (6.0–8.0)	.77	-	-
Cesarean section, *n* (%)	7 (87.5%)	12 (48%)	.10	-	-
Multiple gestation, *n* (%)	1 (12.5%)	3 (12.0%)	.00	-	-
Antenatal corticosteroids, *n* (%)	7 (87.5%)	20 (80%)	.00	-	-
IUGR, *n* (%)	2 (25.0%)	4 (16.0%	.62	-	-
PPROM, *n* (%)	0 (0%)	5 (20%)	.30	-	-
Clinical chorioamnionitis, *n* (%)	0 (0%)	0 (0%)	-	-	-
Antepartum bleeding, *n* (%)	0 (0%)	0 (0%)	-	-	-
Oligohydramnios, *n* (%)	0 (0%)	0 (0%)	-	-	-
Preeclampsia, *n* (%)	3 (37.5%)	5 (20%)	.37	-	-

The table shows the patient characteristics of the sub-cohorts with and without late-onset PH. Data are presented, depending on the distribution of the variable, as mean ± SD, median and interquartile range, or frequencies with percentages. Odds ratios are shown for the variables with a significant association with late-onset PH. *IUGR*, intra-uterine growth retardation; *PPROM*, prolonged premature rupture of membranes; *PH*, pulmonary hypertension; *OR*, odds ratio; *CI*, confidence interval

**Table 4:** Prevalence of PH

	Infants echocardiographically assessed for PH
First 2 weeks after birth	*n* = 118 out of 182
Early-onset PH	46 (39%)
PPHN, *n* (%)	13 (11%)
Flow PH, *n* (%)	33 (28%)
Intermediate period	*n* = 77 out of 163
Flow PH, *n* (%)	13 (17%)
Later-onset PH, *n* (%)	4 (6%)
≥ 36 weeks PMA	*n* = 33 out of 153
Late-onset PH, *n* (%)	8 (24%)
Infants with BPD	*n* = 19 out of 53
Late-onset PH, *n* (%)	5 (26%)
Infants without BPD	*n* = 14 out of 100
Late-onset PH, *n* (%)	3 (21%)

The table shows the prevalence of PH per subtype of PH per time slot. The intermediate period is defined as the period between the other two time slots: 2 weeks after birth and before the age of 36 weeks PMA. *PH*, pulmonary hypertension; *PPHN*, persistent pulmonary hypertension of the neonate; *BPD*, bronchopulmonary dysplasia

Infants with BPD had significantly lower gestational ages, birthweight, and Apgar scores in comparison to infants without BPD. Also, infants with BPD suffered more often from IUGR or maternal preeclampsia (Table 2). Infants with late-onset PH had lower birthweight in comparison to infants without late-onset PH (Table 3).

The presence of PH during the first 2 weeks after birth was associated with the subsequent development of BPD. The odds ratio (OR) was 3.2 (95% confidence interval (CI), 1.4–7.3, *P* = .007). This association persisted in bivariate regression analysis after adjusting for gestational age, birthweight, or the existence of an PDA. We found no association between the presence of early-onset PH and the development of PH at 36 weeks PMA. The OR was 3.3 (95% CI, 0.48–22.0), *P* = .23).

**Fig. 2:**
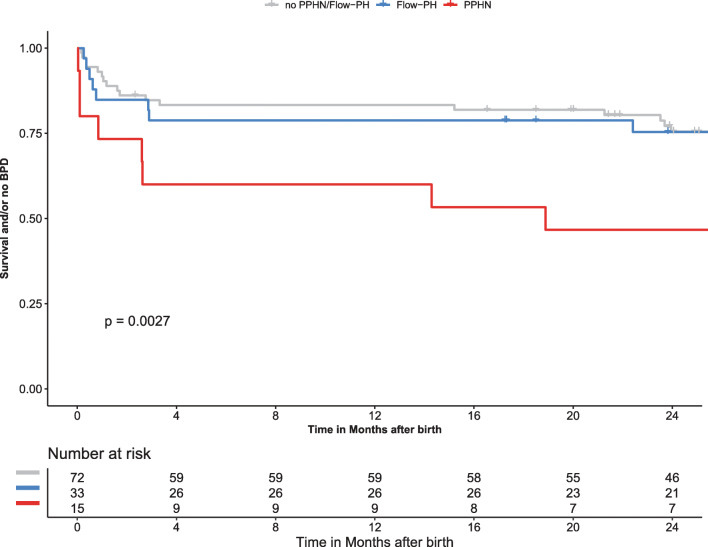
Kaplan-Meier curves showing the association of early-onset PH and survival and/or the development of BPD. The curves of the infants with PPHN, flow PH, and infants without PPHN/flow PH are shown. A log-rank test showed a significant difference with *P* = .003. BPD, bronchopulmonary dysplasia; PPHN, persistent pulmonary hypertension of the neonate; PH, pulmonary hypertension

During a 2-year follow-up period, 36 out of the 182 infants died (20%). The survival rates of the study cohort at 1 month, 3 months, and 6 months was 86%, 81%, and 80%, respectively. We found that early PH was associated with worse survival and with the development of BPD and that this was predominantly driven by infants with PPHN (Fig. 2).

In infants who lived up to 36 weeks PMA, subsequent mortality was 11% for infants with BPD and 1% for infants without BPD. No significant difference in survival rate was found between infants with or without late-onset PH.

### Survey among the Dutch NICUs

The response rate to the questionnaires sent to the ten Dutch NICUs was 100%. The questions were answered by one neonatologist representing each NICU. All responses were received 6 months after the survey was sent. The survey was sent in July 2017, and all responses were received on 27-12-2017. The questions asking for quantitative estimates of the prevalence of BPD and PH in extremely preterm infants revealed a high variation in perceived frequencies of occurrence (Table 5). All ten NICUs indicated that infants with a gestational age of less than 32 weeks and/or with a birthweight of less than 1000 g were not routinely assessed by means of echocardiographic imaging. Echocardiography was performed only if there were clinical reasons to do so. Additionally, all centers indicated that if infants were diagnosed with PH, no standardized follow-up procedure using echocardiography was in place to monitor PH, unless clinically necessary. One NICU reported they had started to routinely assess infants suffering from BPD for the presence of late-onset PH for 1 year. This NICU assessed the presence of PH in these infants somewhere between 36 weeks PMA and 2 years of age.

**Table 5:** Estimated number of infants with PH and BPD

NICU	Number of infants admitted per year (*N*)	Percentage of infants who develop BPD (%)	Estimated percentage of infants who develop BPD and PH (%)	Estimated percentage of infants without BPD who develop PH (%)
1	200	35	30	5
2	350	10	1	0
3	200	15	2	1
4	130	12	12	0
5	200	5	0	0
6	150	20	10	0
7	140	28	-	-
8	185	10	20	5
9	280	20	15	3
10	170	30	0	0

The table shows the given estimated numbers reported by the NICU centers. *NICU*, neonatal intensive care unit; *BPD*, bronchopulmonary dysplasia; *PH*, pulmonary hypertension

## Discussion

Emerging evidence has increased the awareness that PH is common in extremely preterm infants and contributes significantly to morbidity and mortality in these infants. The current study in a cohort of extremely preterm infants confirms this frequent occurrence, while at the same time the real-world clinical practice regarding echocardiographic evaluation for PH in this population falls short. Early-onset PH, presenting in the first 2 weeks after birth, was diagnosed in at least 26% of the total cohort and in 39% of those infants assessed by echocardiography. Of the infants with early-onset PH, almost one-third presented as PPHN, while two-thirds presented as PDA-related flow PH. Thirty-five percent of the cohort developed BPD. The presence of early-onset PH correlated with later development of BPD. Only 36% of infants with BPD were echocardiographically assessed for late-onset PH, revealing late-onset PH in 26% of them. These prevalence numbers correspond to those reported in the international literature and recently reviewed and meta-analyzed [10]. The presence of PH in the first 2 weeks of life, independent of phenotype, has shown to be predictive for the development of BPD at 36 weeks PMA. The prevalence of late-onset PH increases sharply with the severity of BPD, up to almost 40% in severe BPD. The same systematic review and meta-analysis demonstrated that the presence of PH in extremely preterm infants, be it early-onset or late-onset, increased the risk of mortality by a factor of 4.7 (95% CI, 2.7–8.3) up to 40% after 2 years of follow-up [10]. In the current retrospective cohort study, we could confirm this increased mortality risk for early-onset PH, but not for late-onset PH, probably due to small numbers.

Risk factors for the development of BPD and for the occurrence of PH in extremely preterm infants have shown important overlap [10]. We found lower gestational age, lower birthweight, and lower Apgar scores to be associated with BPD, while only lower birthweight was associated with late-onset PH in infants with BPD. This supports earlier observations that intra-uterine growth retardation, low birthweight, and small for gestational age are important risk factors for the development of late-onset PH, indicating that prenatal vascular insults are involved in this pulmonary vascular disease.

In the current study, only 22% of the infants at 36 weeks PMA, and only 19 out of 53 infants with BPD (36%) had been echocardiographically assessed to detect PH. Taking into account the reported prevalence of late-onset PH in this population and its devastating effects on morbidity and mortality, these low proportions of infants who were evaluated for PH by echocardiography are concerning. Echocardiographic screening in the first weeks of life to detect early-onset PH can contribute to an early risk stratification for the development of BPD. Treatment strategies in the NICU already focus on preventing BPD by adjusting ventilation settings, accepting lower saturation limits, and considering treatment with caffeine or corticosteroids. Nevertheless, early identification of infants who are at high risk of developing BPD provides an additional opportunity for a more personalized approach, while at the same time it defines a research population for new therapies to prevent BPD.

Infants with moderate to severe BPD are at high risk for developing late-onset PH that seriously affects outcome, justifying active detection of PH in this population. Since BPD and PH are evolving conditions over time, one should be aware that PH can also present before 36 weeks PMA. Over 20% of infants who were echocardiographically assessed between 2 weeks after birth and 36 weeks PMA appeared to have PH, while 5% was associated with high PVR. Early treatment of PH in these infants, before clinical deterioration due to progressive heart failure or PH crises during intercurrent airway infection, is believed to improve outcome. In addition to advanced (non-) invasive ventilatory care and treatment of triggering conditions, such as gastro-enteral reflux disease, PH-targeted drugs are frequently used in current NICU-practice. These drugs include endothelin receptor antagonists, 5-phosphodiesterase inhibitors, and prostacyclin analogues, have been proven effective in pulmonary arterial hypertension (PAH), and can be administered orally, intravenously, and sometimes subcutaneously. Nitric oxide or nebulized iloprost are administered by inhalation, also per non-invasive ventilation in order to prevent further deterioration and lung injury. Although these compounds are frequently used in current clinical practice, controlled trials showing improved outcomes in late-onset PH are lacking [27–31].

Considering the risk of PH in extremely preterm infants over time, its association with outcome and the potential to modify outcome by early treatment, standardized echocardiographic screening for PH is justified in this population. The remaining question is at what point in time this screening should be offered. At present, it may be too early to recommend early echocardiographic screening (in the first 2 weeks of life) of all preterm infants with a gestational age of less than 30 weeks since therapeutic consequences are insufficiently clear. However, the significant influence of late-onset PH on morbidity and mortality of extremely preterm infants does justify echocardiographic screening of patients at high risk, such as infants with BPD. Early detection of late-onset PH should lead to active detection and potentially treatment of triggering comorbidities, such as aspiration, gastroesophageal reflux, respiratory diseases, pulmonary vein stenoses, or aortopulmonary collaterals. Also, this could lead to the adjustment of general management, such as increasing the lower limit of acceptable oxygen saturation, preventing periods of hypoxemia more rigorously, as well as setting up empirical PH-targeted therapies, and monitoring these infants closely.

Recommendations to screen these preterm infants for late-onset PH have appeared in the literature. For example, the Pediatric Pulmonary Hypertension Network (PPHNet), an interactive multidisciplinary group of PH experts involved in ten North American PH programs, recommends screening and follow-up of preterm infants at 36 weeks PMA and proposed an algorithm based on the severity of BPD and symptoms. Preterm infants suffering from moderate or severe BPD are recommended to be assessed by echocardiography at 36 weeks PMA. When no PH is detected but a persistent oxygen demand exists, the infant should be followed at 4- to 6-month intervals. Infants with mild BPD or no BPD are recommended to be screened only if their clinical condition deteriorates and/or oxygen demand becomes persistent [32].

As a screening technique suitable for all types of PH in preterm infants, echocardiography has its limitations. The criteria and definitions for diagnosing PH should be standardized and, fortunately, international consensus is currently emerging regarding this issue [23, 32–34].

Our survey among the Dutch NICUs showed that the awareness for the condition PH in extremely preterm infants and its consequences was highly variable and confirmed that standardized echocardiographic screening for PH in extremely preterm infants, with or without BPD, was not standard practice in the Netherlands. On the basis of the data currently available on the prevalence of PH in extremely preterm infants and its effect on morbidity and mortality, the authors advocate consensus on a policy regarding echocardiographic screening of particular groups of infants at risk, such as infants suffering from moderate or severe BPD at 36 weeks PMA. The policy agreed upon should urgently be incorporated in up-to-date guidelines regarding BPD and neonatal outpatient follow-up.

Echocardiographic screening for early-onset PH in the first 2 weeks after birth would be useful in further exploring the relationship of this type of PH with the development of BPD and late-onset PH. Moreover, new treatment options and preventive measures against BPD for this group of infants could be investigated in high-risk patients, instead of the current generalized approach. However, this should be in a research setting only.

The retrospective design of the current study has associated limitations. Twenty-three of 182 infants did not complete the 2-year follow-up, potentially leading to underestimation of PH incidence. The absence of systematic echocardiographic screening for PH in all included infants prohibits the determination of an actual prevalence of PH since cases with PH may be missed, while echocardiographic assessment at the discretion of the physician may have resulted in selection bias of infants with the most severe PH. However, the design of the study allowed for the evaluation of the real-world clinical practice of echocardiographic assessment of PH in the population of extreme preterm infants in the Netherlands and demonstrates that coverage is low.

## Conclusion

Pulmonary hypertension is a frequent complication in preterm infants that seriously affects the outcome. At the same time, the real-world clinical practice regarding echocardiographic monitoring for PH in this population falls short. Early-onset PH, occurring during the first 2 weeks after birth, is associated with the subsequent development of BPD at 36 weeks PMA and associated with poor survival. Current European guidelines for BPD and neonatal outpatient follow-up lack clear recommendations for systematic, uniform screening, and follow-up of PH in preterm infants, with or without BPD. Based on current evidence and insights, the authors call for specific guidelines for standardized echocardiographic screening of PH in preterm infants suffering from moderate to severe BPD at 36 weeks PMA. In addition, greater clarity should be obtained regarding the prevalence of early-onset PH and new preventive measures and treatment options need to be investigated to combat BPD and PH.

## Supplementary information

ESM 1 (DOCX 156 kb)

## Data Availability

Data are available on reasonable request.

## Abbreviations

BPD: Bronchopulmonary dysplasia
CI: Confidence interval
FiO_2_: Fraction of inspired oxygen
IUGR: Intra-uterine growth restriction
IVH: Intraventricular hemorrhage
IQR: Interquartile range
LVDD: Left ventricular diastolic dysfunction
NEC: Necrotizing enterocolitis
NICU: Neonatal intensive care unit
NVK: Nederlandse Vereniging voor Kindergeneeskunde [Netherlands Pediatric Society]
OFO: Open foramen ovale
OR: Odds ratio
PAP: Pulmonary artery pressure
PDA: Patent ductus arteriosus
PH: Pulmonary hypertension
PMA: Postmenstrual age
PPHN: Persistent pulmonary hypertension of the newborn
PPHNet: Pediatric Pulmonary Hypertension Network
PPROM: Prolonged premature rupture of membranes
PVS: Pulmonary vein stenosis
ROP: Retinopathy of prematurity
RVSP: Right ventricular systolic pressure
SBP: Systolic blood pressure
tcSO_2_: Transcutaneous oxygen saturation
UMCG: University Medical Center Groningen

## References

[1] Stoll BJ, Hansen NI, Bell EF, Walsh MC, Carlo WA, Shankaran S, Laptook AR, Sánchez PJ, van Meurs K, Wyckoff M, Das A, Hale EC, Ball MB, Newman NS, Schibler K, Poindexter BB, Kennedy KA, Cotten CM, Watterberg KL, D'Angio CT, DeMauro S, Truog WE, Devaskar U, Higgins RD, Eunice Kennedy Shriver National Institute of Child Health and Human Development Neonatal Research Network (2015). Trends in care practices, morbidity and mortality of extremely preterm neonates, 1993-2012. JAMA..

[2] Aarnoudse-Moens CSH, Rijken M, Swarte RM (2017). Follow-up na 2 jaar van kinderen geboren bij 24 weken. Ned Tijdschr Geneeskd.

[3] Northway WH, Rosan RC, Porter DY (1967). Pulmonary disease following respirator therapy of hyaline-membrane disease. Bronchopulmonary dysplasia. N Engl J Med.

[4] Jobe AH, Bancalari E (2001). Bronchopulmonary dysplasia. Am J Respir Crit Care Med.

[5] Bancalari E, Claure N (2006). Definitions and diagnostic criteria for bronchopulmonary dysplasia. Semin Perinatol.

[6] Nederlandse Verenging voor Kindergeneeskunde (NVK). Richtlijn BPD. December 2013.

[7] Steinhorn R, Davis JM, Göpel W, Jobe A, Abman S, Laughon M, Bancalari E, Aschner J, Ballard R, Greenough A, Storari L, Thomson M, Ariagno RL, Fabbri L, Turner MA, International Neonatal Consortium (2017). Chronic pulmonary insufficiency of prematurity: developing optimal endpoints for drug development. J Pediatr.

[8] Mourani PM, Sontag MK, Younoszai A, Miller JI, Kinsella JP, Baker CD, Poindexter BB, Ingram DA, Abman SH (2015). Early pulmonary vascular disease in preterm infants at risk for bronchopulmonary dysplasia. Am J Respir Crit Care Med.

[9] Bhat R, Salas AA, Foster C, Carlo WA, Ambalavanan N (2012). Prospective analysis of pulmonary hypertension in extremely low birthweight infants. Pediatrics..

[10] Arjaans S, Zwart EAH, Ploegstra MJ, Bos AF, Kooi EMW, Hillege HL, Berger RMF (2018) Identification of gaps in the current knowledge on pulmonary hypertension in extremely preterm infants: a systematic review and meta-analysis. Paediatr Perinat Epidemiol 32(3):258–267. 10.1111/ppe.12444.F 10.1111/ppe.12444 29341209

[11] Berger RM, Beghetti M, Humpl T (2012). Clinical features of paediatric pulmonary hypertension: a registry study. Lancet..

[12] Rosenzweig EB, Abman SH, Adatia I, Beghetti M, Bonnet D, Haworth S, Ivy DD, Berger RMF (2019). Paediatric pulmonary arterial hypertension: updates on definition, classification, diagnostics and management. Eur Respir J.

[13] Mirza H, Ziegler J, Ford S (2014). Pulmonary hypertension in preterm infants: prevalence and association with bronchopulmonary dysplasia. J Pediatr.

[14] Nakanishi H, Suenaga H, Uchiyama (2018). Persistent pulmonary hypertension of the newborn in extremely preterm infants: a Japanese cohort study. Arch Dis Child Fetal Neonatal Ed.

[15] Khemani E, McElhinney DB, Rhein L (2007). Pulmonary artery hypertension in formerly preterm born infants with bronchopulmonary dysplasia: clinical features and outcomes in the surfactant era. Pediatrics..

[16] Cuna A, Kandasamy J, Sims B (2014). B-type natriuretic peptide and mortality in extremely low birthweight infants with pulmonary hypertension: a retrospective cohort analysis. BMC Pediatr.

[17] Del Cerro MJ, Sabate Rotes A, Carton A (2014). Pulmonary hypertension in bronchopulmonary dysplasia: clinical findings, cardiovascular anomalies and outcomes. Pediatr Pulmonol.

[18] Arjaans S, Haarman MG, Roofthooft MTR et al The fate of pulmonary hypertension associated with bronchopulmonary dysplasia beyond 36 weeks postmenstrual age. Arch Dis Child Fetal Neonatal Ed In press 2020 10.1136/archdischild-2019-318531 PMC7788204 32571832

[19] Duijts L, van Meel ER, Moschino L, Baraldi E, Barnhoorn M, Bramer WM, Bolton CE, Boyd J, Buchvald F, del Cerro MJ, Colin AA, Ersu R, Greenough A, Gremmen C, Halvorsen T, Kamphuis J, Kotecha S, Rooney-Otero K, Schulzke S, Wilson A, Rigau D, Morgan RL, Tonia T, Roehr CC, Pijnenburg MW (2020). European Respiratory Society guideline on long term management of children with bronchopulmonary dysplasia. Eur Respir J.

[20] Harris PA, Taylor R, Thielke R, Payne J, Gonzalez N, Conde JG (2009). Research electronic data capture (REDCap) – a metadata-driven methodology and workflow process for providing translational research informatics support. J Biomed Inform.

[21] Fuloria M, Aschner JL (2017). Persistent pulmonary hypertension of the newborn. Semin Fetal Neonatal Med.

[22] Singh Y, Tissot C, Fraga MV, Yousef N, Cortes RG, Lopez J, Sanchez-de-Toledo J, Brierley J, Colunga JM, Raffaj D, da Cruz E, Durand P, Kenderessy P, Lang HJ, Nishisaki A, Kneyber MC, Tissieres P, Conlon TW, de Luca D (2020). International evidence-based guidelines on Point of Care Ultrasound (POCUS) for critically ill neonates and chilcren issued by the POCUS Working Group of the European Society of Paediatric and Neonatal Intensive Care (ESPNIC). Crit Care.

[23] Abman SH, Hansmann G, Archer SL, Ivy DD, Adatia I, Chung WK, Hanna BD, Rosenzweig EB, Raj JU, Cornfield D, Stenmark KR, Steinhorn R, Thébaud B, Fineman JR, Kuehne T, Feinstein JA, Friedberg MK, Earing M, Barst RJ, Keller RL, Kinsella JP, Mullen M, Deterding R, Kulik T, Mallory G, Humpl T, Wessel DL, American Heart Association Council on Cardiopulmonary, Critical Care, Perioperative and Resuscitation; Council on Clinical Cardiology; Council on Cardiovascular Disease in the Young; Council on Cardiovascular Radiology and Intervention; Council on Cardiovascular Surgery and Anesthesia; and the American Thoracic Society (2015). Pediatric pulmonary hypertension. Guidelines From the American Heart Association and American Thoracic Society. Circulation..

[24] IBM Corp. Released 2015. IBM SPSS Statistics for Windows, Version 23.0. Armonk, NY: IBM Corp.

[25] RStudio Team (2015). RStudio: integrated development for R. RStudio, Inc., Boston, MA URL http://www.rstudio.com/. [computer program].

[26] R: A Language and environment for statistical computing. R Foundation on for Statistical Computing, Vienna, Austria. [computer program]. 2018.

[27] Kelly LE, Ohlsson A, Shah PS (2017) Sildenafil for pulmonary hypertension in neonates. Cochrane Database Syst Rev 8. 10.1002/14651858.CD005494.pub4 10.1002/14651858.CD005494.pub4 PMC6483280 28777888

[28] Perez KM, Laughon M (2015). Sildenafil in term and premature infants: a systematic review. Clin Ther.

[29] Barrington KJ, Finer N, Pennaforte T, Altit G, Cochrane Neonatal Group (2017) Nitric oxide for respiratory failure in infants born at or near term. Cochrane Database Syst Rev 1. 10.1002/14651858.CD000399.pub3 10.1002/14651858.CD000399.pub3 PMC6464941 28056166

[30] Piastra M, De Luca D, Pia De Carolis M (2012). Nebulized iloprost and nononvasive respiratory support for impending hypoxaemic respiratory failure in formerly preterm infants: a case series. Pediatr Pulmonol.

[31] Mulligan C, Beghetti M (2012). Inhaled Iloprost for the control of acute pulmonary hypertension in children: a systemtic review. Pediatr Crit Care Med.

[32] Krishnan U, Feinstein JA, Adatia I (2017). Evaluation and management of pulmonary hypertension in children with bronchopulmonary dysplasia. J Pediatr.

[33] Mourani PM, Sontag MK, Younoszai A, Ivy DD, Abman SH (2008). Clinical utility of echocardiography for the diagnosis and management of pulmonary vascular disease in young children with chronic lung disease. Pediatrics.

[34] Levy PT, Patel MD, Choudry S (2018). Evidence of echocardiograhic markers of pulmonary vascular disease in asymptomatic infants born preterm at one year of age. J Pediatr.

