# Supplementary material for: Pulmonary hypertension in extremely preterm infants: a call to standardize echocardiographic screening and follow-up policy
Source: Eur J Pediatr. 2021 Feb 2;180(6):1855–65. doi: 10.1007/s00431-021-03931-5 (PMC8105237; doi:10.1007/s00431-021-03931-5)
Supplement: Supplementary file 1 — (DOCX 156 kb) [file 431_2021_3931_MOESM1_ESM.docx]

**Pulmonary hypertension in extremely preterm infants. A call to standardize echocardiographic screening and follow-up policy**

Sanne Arjaans^1^ , s.arjaans@umcg.nl, Elvira. A.H. Zwart^1^, e.a.h.zwart@student.rug.nl, Marc Roofthooft^1^, m.t.r.roofthooft@umcg.nl, Elisabeth M.W. Kooi^2^, e.m.w.kooi@umcg.nl, Arend F. Bos^2^, a.f.bos@umcg.nl, Rolf M.F. Berger^1^, r.m.f.berger@umcg.nl

**Supplemental Material**

**Supplementary Figure 1**

**
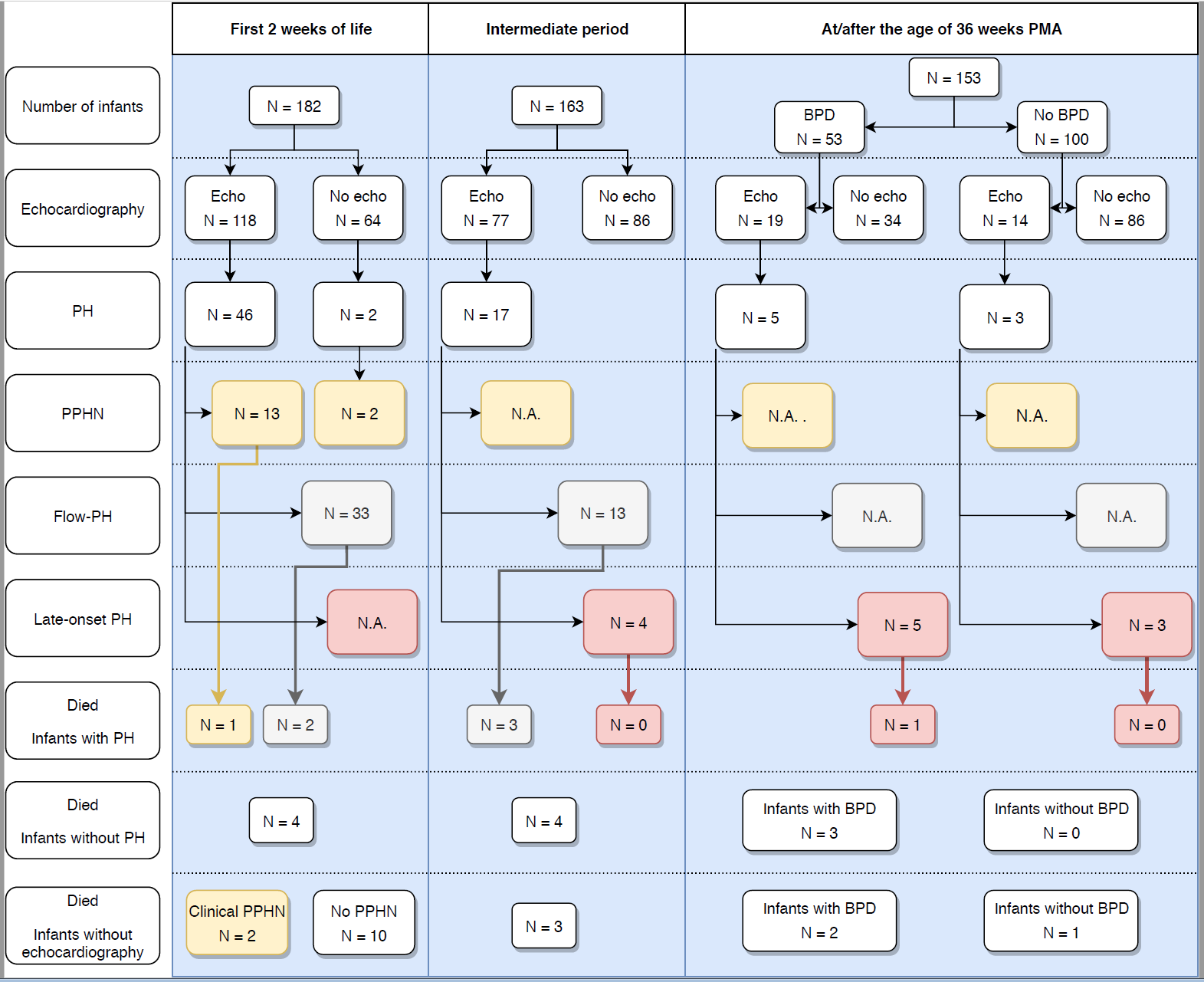
**

**Figure legend:**

Flow chart of all echocardiographs made of the 182 infants included during the study period. The figure is divided into three time slots: The first two weeks after birth, at/after 36 weeks’ PMA, and the intermediate period. Per slot we indicate of how many infants an echocardiograph was made and how many of these infants have PH and which PH phenotypes they developed. In two infants during the first two weeks after birth, PPHN was clinically diagnosed based on a preductal and postductal tcSO_2_ difference of more than 10%.

The intermediate period is defined as the period between the other two time slots: After two weeks after birth and before the age of 36 weeks PMA.

Abbreviations: PH, pulmonary hypertension; BPD, bronchopulmonary dysplasia; PPHN, persistent pulmonary hypertension of the neonate, PMA, postmenstrual age.

**Supplementary Table 1. Patient characteristics of surviving infants divided per total months of follow-up.**

| **Variable** | **Infant with > 24 months follow-up**  **N = 123** | **Infants with < 24 months follow-up**  **N=23** | |
| --- | --- | --- | --- |
| **Sex (male)** | 64 (52.0%) | 11 (47.8%) | |
| **Gestational age, weeks** | 27.7 ± 1.2 | 27.9 ± 1.4 | |
| **Birthweight, g** | 1074.0 ± 279.4 | 1066.7 ± 230.1 | |
| **Apgar score, 5th minute** | 7.0 (6.0 – 8.0) | 7.0 (6.0 – 8.0) | |
| **Cesarean section, n (%)** | 63 (51.2%) | 14 (60.8%) | |
| **Multiple gestation, n (%)** | **27 (22.0%)^#^** | **10 (43.5%)^#^** | |
| **Antenatal corticosteroids** | 103 (83.7%) | 17 (73.9%) | |
| **IUGR, n (%)** | 24 (19.5%) | 3 (13.0%) | |
| **PPROM, n (%)** | 21 (17.1%) | 1 (4.3%) | |
| **Clinical chorioamnionitis** | **0 (0.0%)^#^** | **2 (8.7%)^#^** | |
| **Antepartum bleeding, n (%)** | 11 (8.9%) | 1 (4.3%) | |
| **Oligohydramnios, n (%)** | 6 (4.9%) | 2 (8.7%) | |
| **Preeclampsia, n (%)** | 15 (12.2%) | 2 (8.7%) | |
|  |  |  |  |

Patient characteristics of the surviving infants divided per total months of follow-up are shown.

Data are presented, depending on the distribution of the variable, as mean±SD, median and interquartile range or frequencies with percentages.

Abbreviations: IUGR, intra-uterine growth retardation; PPROM, prolonged premature rupture of membranes

^#^Significant difference between the three groups.
